# Toward Advanced Nursing Practice along with People-Centered Care Partnership Model for Sustainable Universal Health Coverage and Universal Access to Health^1^

**DOI:** 10.1590/1518-8345.1657.2839

**Published:** 2017-01-30

**Authors:** Tomoko Kamei, Keiko Takahashi, Junko Omori, Naoko Arimori, Michiko Hishinuma, Kiyomi Asahara, Yoko Shimpuku, Kumiko Ohashi, Junko Tashiro

**Affiliations:** 2 PhD, Professor, St. Luke’s International University, Graduate School of Nursing, WPRO/WHO Collaborating Center for Nursing & Midwifery for Development of People-Centered Care in Primary Health Care, Tokyo, Japan.; 3 PhD, Associate Professor, St. Luke’s International University, Graduate School of Nursing, WPRO/WHO Collaborating Center for Nursing & Midwifery for Development of People-Centered Care in Primary Health Care, Tokyo, Japan.; 4 PhD, Professor, Tohoku University, Graduated School of Medicine, Miyagi, Japan.; 5 PhD, Professor, Niigata University, School of Health Sciences, Faculty of Medicine, Niigata, Japan.; 6 PhD, Assistant Professor, St. Luke’s International University, Graduate School of Nursing, WPRO/WHO Collaborating Center for Nursing & Midwifery for Development of People-Centered Care in Primary Health Care, Tokyo, Japan.

**Keywords:** Advanced Practice Nursing, Nursing Care, Delivery of Health Care, Nursing

## Abstract

**Objective:**

this study developed a people-centered care (PCC) partnership model for the aging society to address the challenges of social changes affecting people’s health and the new role of advanced practice nurses to sustain universal health coverage.

**Method:**

a people-centered care partnership model was developed on the basis of qualitative meta-synthesis of the literature and assessment of 14 related projects. The ongoing projects resulted in individual and social transformation by improving community health literacy and behaviors using people-centered care and enhancing partnership between healthcare providers and community members through advanced practice nurses.

**Results:**

people-centered care starts when community members and healthcare providers foreground health and social issues among community members and families. This model tackles these issues, creating new values concerning health and forming a social system that improves quality of life and social support to sustain universal health care through the process of building partnership with communities.

**Conclusion:**

a PCC partnership model addresses the challenges of social changes affecting general health and the new role of advanced practice nurses in sustaining UHC.

## Introduction

Advanced nursing practices are new aspect in Japan; nurses are facing a historical turning point in their professional role, derived from a combination of internal professionalizing drivers, external political intentions, and social needs^1^ ^-^ ^3^. Nurses are expected to become agents of change in the health system and expand their role into a new and uncharted territory with advanced skills, knowledge, and competencies. In health system, nurses must transform the traditional scope of nursing practice and institutional bedside care into new nursing fields in each country. Effective partnership with and participation of nurses will contribute to universal health coverage (UHC)^4^.

The increased number of non-communicable diseases (NCDs) constitutes a social need. Currently cardiovascular diseases account for the most NCD deaths, i.e., 17.5 million people annually, followed by cancer (8.2 million), respiratory diseases (4 million), and diabetes (1.5 million). These four groups of diseases account for 82% of all NCD deaths worldwide (WHO, 2015)^5^. In addition, NCD patients need lifelong health care services and large health care expenditures. To suppress the financial constraints of health care, it is important to improve lifestyles to prevent NCDs and promote health, specifically enhancing health literacy for all ages.

UHC is firmly based on the World Health Organization (WHO) constitution of 1948, which declares health to be a fundamental human right. The Health for All agenda was set in the Alma-Ata declaration in 1978. Equity is paramount, which means that countries need to track progress not only simply over the national population but also within groups differentiated by income level, sex, age, residence, and ethnicity^6^. The goal of UHC is equity in access to health services, implying that the quality of health services is ensured and that all people obtain the health services they need without financial hardship^6^. To achieve UHC, each community needs a strong, efficient, and well-run health system meeting the important social needs. In Japan, the health care insurance system and the long-term care insurance system were established in 1961 and 2000 respectively, and these were spread as a public UHC in accordance with rapidly increasing the proportion older adults and shrinking family members in a house hold. Therefore it is necessary to promote not only disease prevention but also health services specifically in the local residential community. In such a system, it is essential to ensure people-centered integrated health care and affordability, meaning a system for financing health services so that people do not suffer financial hardship when using them, as well as access to essential medicines and technologies to diagnose and treat medical problems and a sufficient capacity of well-trained and motivated health workers to provide the services to meet patients’ needs on the basis of the best available evidence^6^.

In September 2015, at the General Assembly of the United Nations, the 2030 Agenda for Sustainable Development^7^ was adopted as new common goals of the international community to achieve by 2030 in order to tackle the remaining challenges after the Millennium Development Goals. In the context of UHC, nurses could contribute by continuing education and strengthening their profession, making effective partnerships, and providing significant leadership and innovation to society^4^. This means that nurses are expected to create partnership with community members, rather than simply providing technical assistance within medical institutions.

People-centered care (PCC) is a process of health and nursing care that enhances health literacy as well as motivates community members of all ages to seek their own health care; thus, healthcare providers, especially nurses, must help community members make decisions in partnership^8^. People-Centered Health Care is also a special initiative in the WHO Western Pacific Region; it is an umbrella term that better encapsulates the foremost consideration of the patient across all levels of health systems^9^. This supports the process in which community members address their own health challenges as well as those of society. Since 2003, our institution of World Health Organization Collaborating Center (WHO CC) has been organizing PCC projects for community members of all ages; it has the accumulated knowledge to develop the PCC partnership model as an advanced nursing practice^10^.

PCC initiatives have been given additional urgency by the issue of aging, which Japan and other countries are currently suffering from. The life expectancies of Japanese males (80.5 years) and of Japanese females (86.8 years) (Ministry of Health, Labour and Welfare, 2015)^11^, are the one of longest life expectancies in the world for both sexes (Ministry of Health, Labour and Welfare, 2013)^12^. However, Japan also faces a low birth rate, which means a decreasing population and an ongoing shortage of healthcare providers and social security. Older adults also tend to require medical care at high expenditures^13^. In Japan, medical expenses for individuals of more than 75 years of age reach 4.41 times that for those less than 75 years^14^. Moreover, the increasing proportion of older adults aged 65 and over (26.7%)^15^ has resulted in a greater number of cases of neuro-cognitive disorder and dementia^16^. In Japan, as a country that has achieved UHC, health care access is easy regardless of necessity, which has dramatically increased medical expenses. In this context, health literacy must be improved by forming a partnership between community members and healthcare providers to efficiently improve health as well as to reduce unnecessary medical expenses so that those in need can properly access health care.

This study develops a PCC partnership model in an aging society to address the challenges of social changes affecting general health and the new role of advanced practice nurses in sustaining UHC.

## Method

This study develops the PCC partnership model in two steps. The first step is a literature review of the concept of PCC and a practice-based assessment of our WHO CC PCC projects. The second is a qualitative meta-synthesis of both results of the literature review and the projects and the results of participants’ satisfaction for the construction of a PCC partnership model.

### Literature search strategies

English databases were used for this study, including the Cumulative Index of Nursing and Allied Health Literature (CINHAL); CINHAL Plus with Full Text (EBSCO); MAGAZINEPLUS; PubMed; EMBASE; Nursing & Allied Health Source (NAHS); and the Japanese published medical work database, including Japan Medical Abstracts Society (JAMAS) and Citation Information by the National Institute of Informatics (CiNii). Both English and Japanese articles were included; no language limitations were imposed. Keyword combinations used in the search were as follows: “people-centered care” or “people-centered health care” or “partnership” and “concept analysis.” Search dates for the above databases were from January 1, 1980 to July 30, 2015. Manual search was performed to identify books, articles and project reports in our institution.

### Article selection, inclusion and extraction parameters

Eligible studies included published work involving PCC and/or partnerships with health care providers and community members. Qualitative, observational, and quasi-experimental studies; pre- and post-implemental studies without comparators; and prospective cohort studies were included. Original articles, research reports, practical reports, and commentaries were reviewed. Published conference proceedings including abstracts of research presented at conferences and article titles without partnership were extracted. Unpublished articles or those not easily accessible via the above databases were not reviewed.

### PCC concept assessment strategies in the articles

The concept of PCC in each study was assessed in three steps. First, one author independently reviewed the abstracts and full texts, applying the selection criteria to identify the concept of PCC. Second, all nine authors reviewed and categorized the full text of the articles. Each article was analyzed using a customized data extraction form, created by the first author, comprising the following: PCC process with resources, partnership, and capacity building; process of change; and outcome of change and health care system for community members, healthcare providers, and community. The researchers discussed the extracted categories until a final consensus was reached.

### Our WHO CC PCC project assessment strategies

The 14 projects of our WHO CC PCC in 2015 targeting community members of all ages were analyzed according to participants, health issues, types of partnerships with community members and healthcare providers, satisfaction, and outcomes. The visual analogue scale (VAS-10) was used to assess participant satisfaction for each project during the fiscal year 2015 (April 2015 to March 2016).

### Conceptualizations and modeling of PCC

The results of both the literature review and the PCC project assessment were integrated by qualitative meta-synthesis^17^. The PCC partnership model was constructed and illustrated using the flow of input, process, and outcomes.

## Results

### PCC concept analyses through literature review

The literature search identified 196 citations. The keyword “partnership” showed 116 citations, 32 for “people-centered care,” and 48 for “people-centered health care.” Of these, 152 were excluded on the basis of extraction criteria. The remaining 42 citations were reviewed: 21 original articles, 10 research reports, eight commentaries on the same projects, two brief reports, and one WHO report, as well as two books, totaling 44 references^18^ ^-^ ^61^.

PCC resources were categorized into easy and expanded access to health services; useful health information for each person; a place in the community to easily gather; and interaction with other people of all ages, health volunteers, and experts such as nurses and counselors. These resources were too convenient to each member who had primary health needs.

Partnerships were major relation pattern among community members, health volunteers, and healthcare providers, involving shared decision making and responsibilities.

Capacity building involved the following capacities of the people involved: acceptance and appreciation of people’s beliefs and worth, healthy behavior and thoughts, health literacy that assists the choice of information appropriate for each person’s health; the understanding of where and how to speak of one’s own health; the capacity and ability to make subjective health behavior or decisions; enduring self-care, self-efficacy, and strength to maintain health; and information communication technology literacy. These capacities led to the improved health and psychological well-being of the people.

The change process involves the following: improvement and changes of health behavior; subjective awareness of oneself; experience being healed by others; setting others’ minds at rest; feelings of comfort; improvement of health consciousness and behaviors, physical states, and emotional well-being; vigor in daily activities; and health volunteers starting a project subjectively.

The outcome of change and the health care system showed increased opportunity to receive health information; creation and spread of sustainable, appropriate services for community members and new values; making a key/core person in the community; collaboration with local government; and shared future targets with community members and healthcare providers through debriefing of each PCC activity. These outcomes show people’s increased interest and motivation to initiate decision making on their own health.

### PCC activities through our WHO CC PCC project

Our institution was first designated as a WHO CC for Nursing Development in Primary Health Care (PHC) in 1990 and has been re-designated six times over the last 24 years. In 2016, our WHO CC began to assist WPRO and member states in the development of community PCC models based on the values of PHC in the context of aging societies. One of our top tasks is the development of a regional action framework to help countries achieve UHC by promoting integrated PCC service delivery. In 2015, the nursing faculty and community collaborators applied PCC concepts to 14 projects at the university building in Tokyo, focusing on the improvement of the health literacy, behavior, and well-being of community members of all ages. The health issues focused on were as various as those pertaining to families expecting children, stillbirths, mothers with small children, women suffering from infertility, adults and older adults with chronic illnesses and NCDs, frail older adults needing fall prevention awareness, older adults with neuro-cognitive disorders and dementia, and caregivers without health information, as well as an intergenerational program for frail or dementia-suffering older adults and school-aged children in the super-aging Japanese society. Our WHO CC provided PCC services through these projects to encourage community members to take initiative of their own health; 4,721 community members participated annually^62^.

The formation of partnerships between community members and healthcare providers was categorized into three patterns, namely, the pattern of the Approaching Partnership, that of the Supporting Partnership, and that of the Collaborating Partnership. The types of partnership change along with the progress of PCC, depending on the characteristics of the health issues and the community’s degree of consciousness toward them. The Approaching Partnership occurs when healthcare providers lead community members; community members in such a partnership are not aware of health or community issues. In the Supporting Partnership, healthcare providers support community members as needed according to their situations, such as diseases or conditions of aging. At the level of the Collaborating Partnership, community members are fully aware of their health or community issues and participate in activities and decision making. The satisfaction of community members, assessed by VAS-10, was at 8.9 points for fiscal year 2015.

### Development of the conceptual model of PCC Partnership

The authors constructed the PCC partnership model using the qualitatively synthesized and integrated findings of research in the literature and PCC research projects. This model is intended to cover various health and social issues concerning community members and their families, create new values concerning health, and form a social system that guarantees quality of care during the building process of partnership between community members of all ages and healthcare providers to sustain UHC.

**Figure 1:**
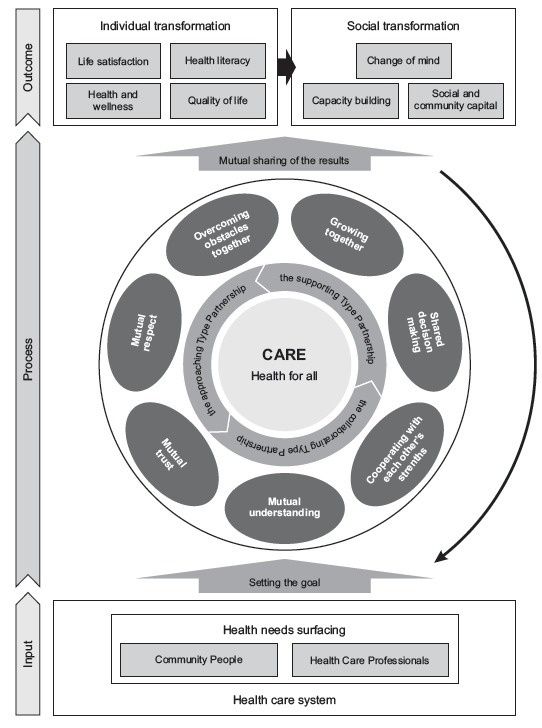
People-Centered Partnership Care Model

The three layers of PCC Process are input, process, and outcome. Input begins when healthcare providers bring health issues to the surface, showing they share aims and goals in health behaviors and resolving problems with community members in their approach to health and social issues particular in their own health. In the next step, healthcare providers and community members build a partnership and setting the goal of health behavior, which will lead to the embodiment of PCC. There are nine attributes in the three layers of PCC: 1) setting goals in health behavior; 2a) mutual understanding (relationship Basis): the community member and healthcare providers understand each other’s strengths, roles, and responsibilities; b) mutual trust (relationship basis): the two trust each other without anxieties; c) mutual respect (relationship basis): the two respect each other in their activities; d) growing together (relationship basis): community members and healthcare providers grow and learn from each other; e) shared decision making (activity approach): the two share the decision-making processes for the same purpose; f) cooperating with each other’s strengths (activity approach): the two make the most of each other’s strengths on an equal footing; g) overcoming obstacles together (activity approach); and 3) mutual sharing of the results of the partnerships: the two overcome obstacles together deriving strength from each other. Through these processes, individual and social transformations are triggered among community members and healthcare providers, sharing achievements with each other as an outcome. In this PCC Process, the Approaching Partnership, the Supporting Partnership, and the Collaborating Partnership were adopted depending on the characteristics of health issues facing community members and the community’s consciousness of health for all (Figure 1).

## Discussion

In this study, the authors developed a PCC partnership model through a literature search and PCC project assessment. The PCC partnership model was structured following the flow of input, process, and outcome. The process includes three types of partnership. The nine attributes of these partnerships were identified as follows: setting goals, mutual respect, mutual trust, mutual understanding, shared decision making, cooperating with the strengths of both, overcoming obstacles together, growing together, and mutual sharing of the results. In the outcome, transformation occurs in individual health needs and social needs. These elements showed a similarity with other former results^24^, which suggest that attitude, respect, and communication were the keys for a partnership between community members and healthcare providers. We found that when people became the main actors for their own health care, it strengthened their mental and physical health. This, then, should contribute to a reduction in soaring medical costs because partnership with healthcare providers can give people strength and improve their health literacy and behavior, which will subsequently promote their health. As the statement of the WHO (2015)^63^ illustrates, the whole picture of PCC includes a political agenda, whereas this model focuses more on the relationship between people and healthcare providers, providing a detailed process of translation among people and within society.

As aging is a serious and common issue throughout the entire developed world and is dramatically becoming prevalent in middle- and low-income countries, our model is expected to provide a new model for enlightening local communities on this issue. Based on its success in our WHO CC projects, it is important to notice the new role of nursing universities in providing not only education but also community nursing services, where anyone can discuss their health issues with nurses literate in research evidence. PCC encourages the growth and development of community members to the point where they will understand that they are the ones responsible for maintaining and improving their own health. Nurses are considered agents of change for health care, and it was thought that these nursing skills; taking partnerships with community members and changing health care, are advanced nursing practices; these new role emerged in response to personal health issues and to external social health policies. The next step for this study is to develop evaluation tools to measure how the PCC partnership model can create social transformation. At the same time, as literature and projects stem mostly from the cases of developed countries, it is necessary to evaluate the applicability of the model to developing countries.

Health systems in an aging society need to be sustainable, strong, and comprehensive. It is essential to provide sufficient human resources so that community members can partner with nurses. Cooperation with nurses and mutual respect will improve quality of life and dignity. Understanding this new role of partnership will enable successful implementation and competencies in nursing leadership to ensure nursing’s contribution to UHC^4^.

## Conclusion

In summary, this study developed a PCC partnership model that emphasizes people’s active participation in health care and the new role of advanced nursing practice. The PCC partnership model aims to tackle various health and social issues with community members and their families, creating new values concerning health and forming a social system guaranteeing quality of life and social capital during the process of building partnerships between community members of all ages to sustain UHC.
